# Supplementary material for: Inequality and fairness with heterogeneous endowments
Source: PLoS One. 2022 Oct 31;17(10):e0276864. doi: 10.1371/journal.pone.0276864 (PMC9621428; doi:10.1371/journal.pone.0276864)
Supplement: S1 Table — The analysis is implemented as a multi-level logistic regression model with random intercepts by group and participant. (PDF) [file pone.0276864.s001.pdf]

	Estimate	Std. Error	*p*-value
Round	−0.042	0.028	0.130
Treatment E	−0.483	0.428	0.260
Treatment EO	−0.824	0.491	0.093
Treatment O	−0.585	0.445	0.188
Endowment = 4	0.482	0.384	0.209
Endowment = 6	−0.241	0.537	0.654
Score	−0.426	0.219	0.052
Intercept	−5.494	0.462	0.000
Participant-level variance	0.000		
Group-level variance	0.000		
Log Likelihood	−253.9		
AIC	527.7		

Note: The model is estimated over 15,891 observations of 777 participants in 40 groups.
