# Supplementary material for: Inequality and fairness with heterogeneous endowments
Source: PLoS One. 2022 Oct 31;17(10):e0276864. doi: 10.1371/journal.pone.0276864 (PMC9621428; doi:10.1371/journal.pone.0276864)
Supplement: S1 Fig — Screenshots from the interactive tutorial (top) and the 19th round of a 17-player game in the treatment with visible endowments (bottom). The current player has an endowment of two but has not allocated their resources to anyone yet. The orange arcs indicate the player’s interactions from the previous round with animations showing the number of resources given or received. The block in front is highlighted because the current player’s cursor is pointing to it. If the player clicks on the block, one of their two resources will transfer to this block. (PDF) [file pone.0276864.s002.pdf]

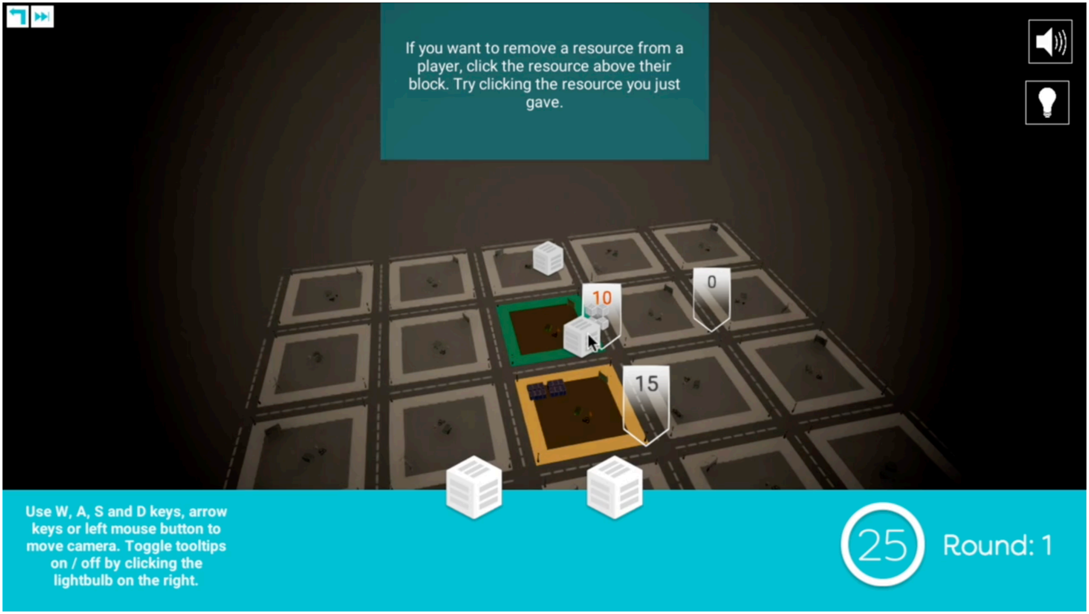
If you want to remove a resource from a player, click the resource above their block. Try clicking the resource you just gave.
10
0
15
Use W, A, S and D keys, arrow keys or left mouse button to move camera. Toggle tooltips on / off by clicking the lightbulb on the right.
25
Round: 1

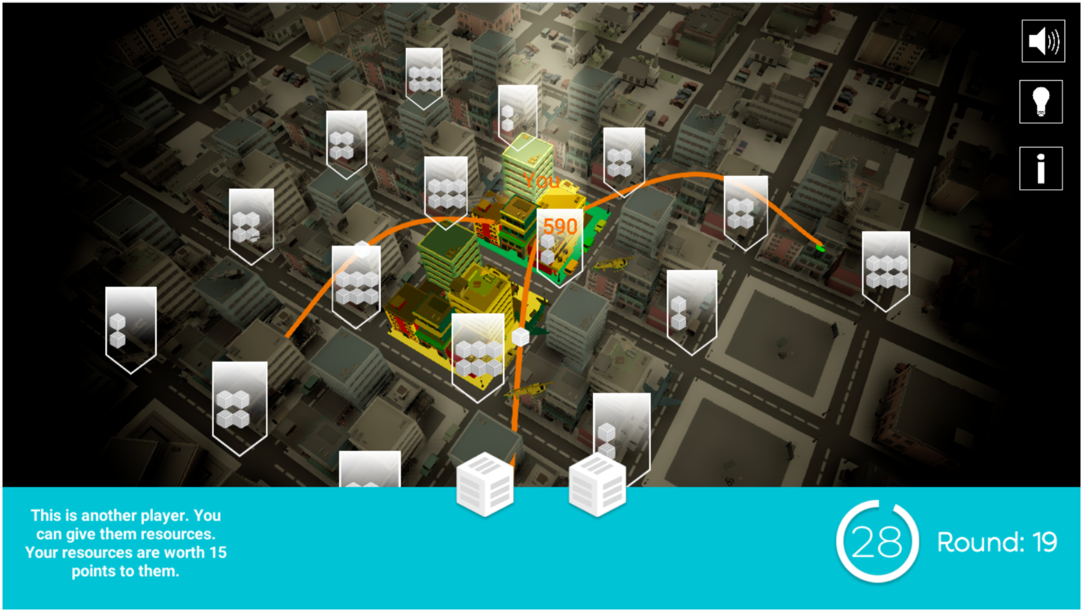
You
590
This is another player. You can give them resources. Your resources are worth 15 points to them.
28
Round: 19
