# Supplementary figures and images for: Inequality and fairness with heterogeneous endowments
Source: PLoS One. 2022 Oct 31;17(10):e0276864. doi: 10.1371/journal.pone.0276864 (PMC9621428; doi:10.1371/journal.pone.0276864)

NI

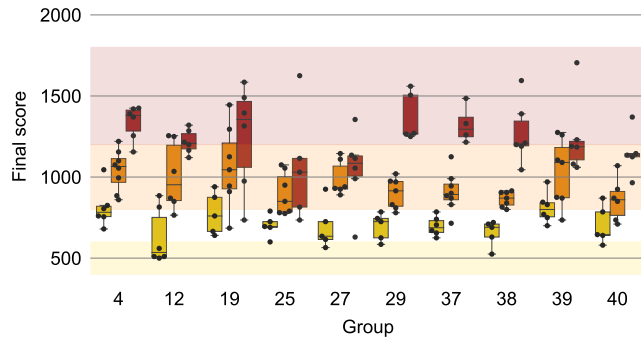

E

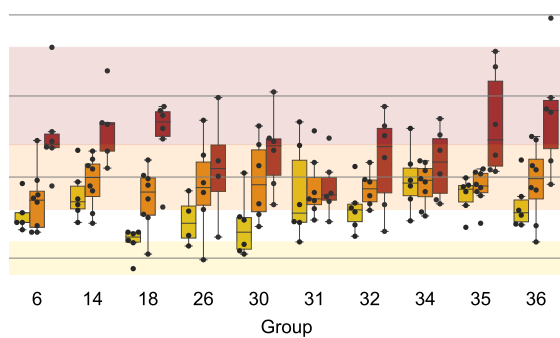

O

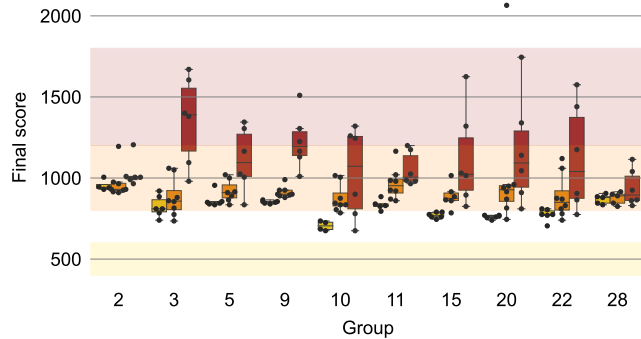

EO

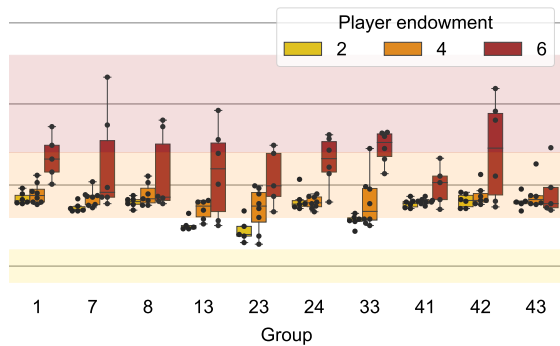

Supplement: S2 Fig — The figure shows boxplots and datapoints of players’ final score depending on their endowment for each group in each treatment. The shaded areas show what score a player of the specified endowment will get if they either do not cooperate at all or invest all their resources in partners who reciprocate perfectly. This is the situation with ci = c−i = 0 and ci = c−i =ri, where ci is the number of resources player i gives to others, c−i is the number of resources other players give to i, and ri is the sum of resources player i has access to over the game. (PDF) [file pone.0276864.s003.pdf]

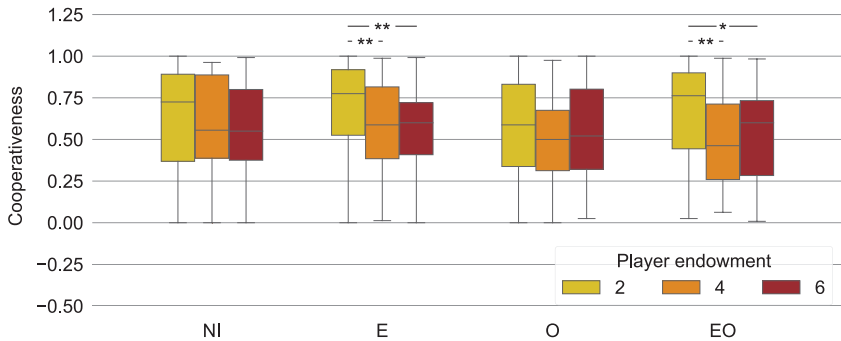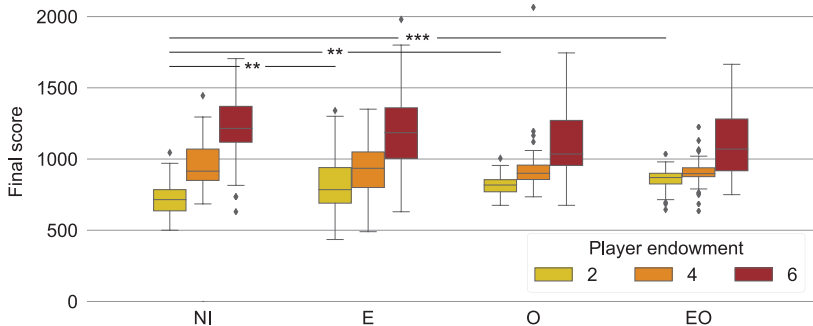

Supplement: S3 Fig — The figures show boxplots of players’ cooperativeness (top) or final score (bottom) for players with different endowments. Cooperativeness is defined as the proportion of available resources invested in others. The asterisk brackets show statistically significant pairwise differences tested in individual-level linear regression models with random intercepts by group: * p<0.05,** p<0.01,*** p<0.001. (PDF) [file pone.0276864.s004.pdf]

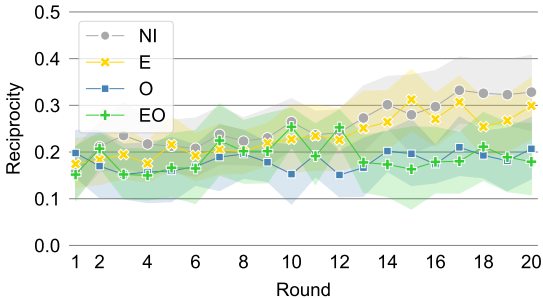

Supplement: S4 Fig — The figure shows the network reciprocity for the groups in the four treatments. Reciprocity is estimated as the ratio of the number of edges pointing in both directions to the total number of edges in the network. The shaded areas correspond to the 95% confidence intervals around the means. (PDF) [file pone.0276864.s005.pdf]

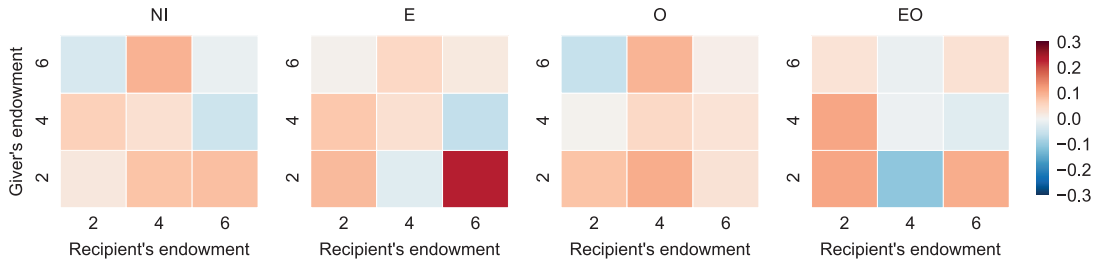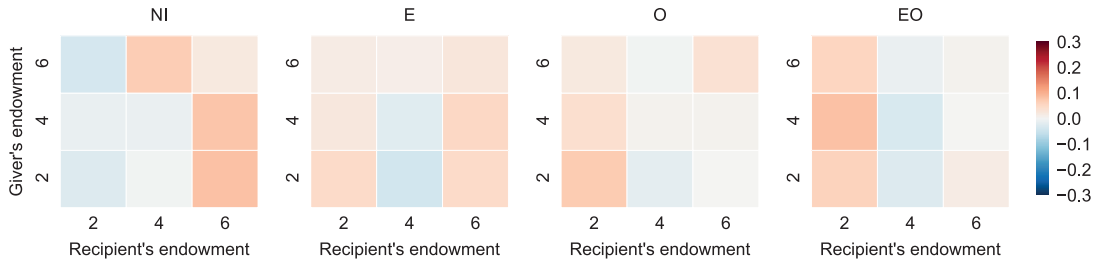

Supplement: S5 Fig — When endowments are visible (E), the least endowed prefer to give to the most endowed in the first round (top) but this charitable giving by endowment does not persist throughout the game (bottom). The heatmaps show the difference between the proportion of resources given in the first round (top) or overall in the game (bottom) by players with the giver’s endowment to players with the recipient’s endowment and the proportion expected to be given if the selected number of resources were allocated at random. (PDF) [file pone.0276864.s006.pdf]

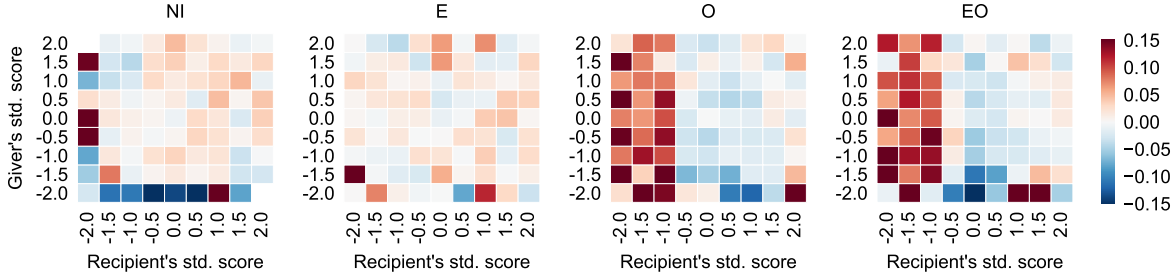

Supplement: S6 Fig — The heatmap shows the difference between the proportion of resources given by players with the giver’s score to players with the recipient’s score and the proportion expected to be given if the resources were allocated at random. The givers’ and recipients’ scores are standardized per game round and the cells show the mean over the 200 game rounds (20 rounds × 10 games per treatment). The highest score values include players with score that is two standard deviations or higher than the mean. The extreme values in the leftmost column and the bottom row are due to the small number of observations of players with score that is two standard deviations below the mean. (PDF) [file pone.0276864.s007.pdf]

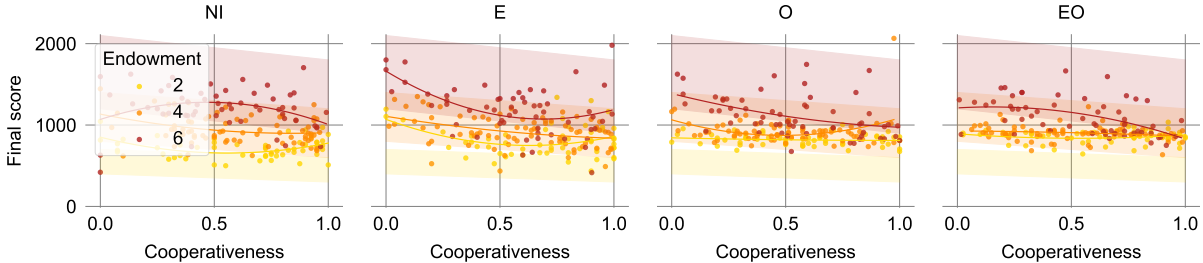

Supplement: S7 Fig — The less dispersed outcomes in the treatments with visible outcomes (O and EO) come at the expense of well-endowed (Endowment = 6) altruistic players. The figure plots players’ scores at the end of the game against their cooperativeness ci, measured as the proportion of resources ri that the player chooses to invest in others. The curves are the best fitting quadratic polynomials for each endowment level. The shaded areas show the expected score for a player who does not cooperate (ci = 0) and receives nothing from others (c−i = 0, bottom left) or receives the equivalent of half of their own resources (c−i =ri/2, top left), and for a player who invests all their own resources in others (ci = ri) and receives exactly the same back (c−i =ri, top right) or just half of that (c−i =ri/2, bottom right). (PDF) [file pone.0276864.s008.pdf]

Endowment 2

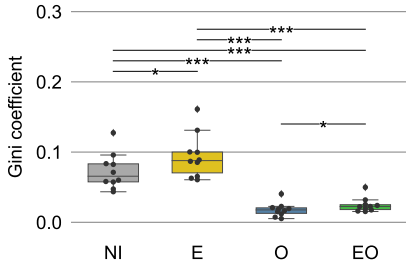

Endowment 4

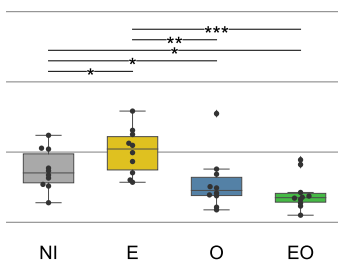

Endowment 6

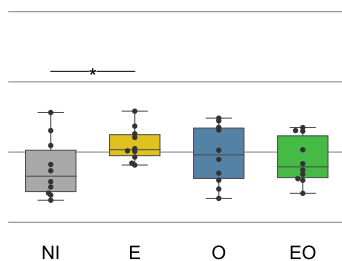

Supplement: S8 Fig — The figure shows the Gini coefficient of outcomes for individuals with the particular endowment by group. The asterisk brackets show statistically significant pairwise differences tested with the Mann-Whitney U test: * p<0.05,** p<0.01,*** p<0.001. (PDF) [file pone.0276864.s009.pdf]
