# Supplementary material for: Inequality and fairness with heterogeneous endowments
Source: PLoS One. 2022 Oct 31;17(10):e0276864. doi: 10.1371/journal.pone.0276864 (PMC9621428; doi:10.1371/journal.pone.0276864)
Supplement: S1 Text — (PDF) [file pone.0276864.s010.pdf]

Game design

The game Urbanizer was developed in collaboration with Science at Home, a citizen science team based at Aarhus University, Denmark. The game is implemented in Unity and runs on the computer (but not mobile) versions of the Chrome, Firefox, and Edge browsers.

In Urbanizer, each player occupies a block in a city and gains points by building and developing their block with resources available in their resource bank or received from other players (S1 Fig). When a player clicks on a block (their own or another player's), one resource is subtracted from their resource bank and gets transferred above the block. If the player clicks on the transferred resource, they undo their action and the resource returns to their resource bank. Players can give multiple resources to a block in each round. Any resources left in the player's resource bank at the end of the round are wasted and do not affect the player's score. Players' blocks are situated on a torus to avoid boundary effects, but to fit the game narrative, each player views the game world as a 4×5 grid in which they occupy a central position. Players with different endowments are distributed on this grid so that every player has a similar mix of differently endowed neighbors.

Participants are instructed to complete the tutorial before joining the waiting room. We include a screenshot from the tutorial in S1 Fig and enclose the full script in the subsection below.

After completing the tutorial, participants join the waiting room. As soon as 16 participants have joined, a three-minute countdown begins such that the game starts as soon as the number of participants reaches 20 or the countdown ends.

The first round lasts 45 seconds and includes an additional pop-up message that informs participants that everyone's starting score is 0, resources per round vary among players, and the specific number of resources the player will receive (see the relevant subsection below for the exact text). Every round after the first lasts 30 seconds. In these rounds, players can observe animated "trade routes" that visualize how many resources they gave to whom and how many resources they received from whom in the previous round.

Script for the tutorial

Welcome to Urbanizer! [Continue]

The goal of the game is to build and develop your city block so that it becomes a major city hub. [Continue]

This is your city block. [Continue]

Every round, you get new resources. You can do two things with a resource: give it to yourself or give it to another player. [Continue]

To give a resource to yourself, simply click on your block.

The resource above your block shows that you have given yourself a resource. [Continue]

Try clicking on another player to give them a resource.

If you want to remove a resource from a player, click the resource above their block. Try clicking the resource you just gave.

When you take a resource back, it returns to your resource bank. [Continue]

At the end of each round, all resources you gave to yourself or other players are used for building. If any resources are left in your bank, they will be lost so make sure to use them! [Continue]

[Next Round in 3…2…1]

This is a trade route. It shows who gave you resources and whom you gave resources to in the previous round. [Continue]

The number above your block shows your score. The boxes show that you get 4 resources each round. [Continue]

Remember, the goal is to become a major city hub, so build and develop your city block as much as possible, however you can! [End Tutorial]

Additional information shown in Round 1

All players start with a score of 0.

Some players get 2 resources each round, some 4, and some 6.

You will get [2, 4, 6] resources each round.
