# Supplementary material for: Inequality and fairness with heterogeneous endowments
Source: PLoS One. 2022 Oct 31;17(10):e0276864. doi: 10.1371/journal.pone.0276864 (PMC9621428; doi:10.1371/journal.pone.0276864)
Supplement: S2 Text — (PDF) [file pone.0276864.s011.pdf]

Temporal exponential random graph models

The temporal exponential random graph model (TERGM) is an extension of the exponential random graph model (ERGM) that accounts for the time dependencies in longitudinally observed networks (Hanneke, Fu, and Xing 2010; Leifeld, Cranmer, and Desmarais 2018).

The standard cross-sectional ERGM predicts the probability of observing a given network with a vector of statistics: $P(N, \boldsymbol{\theta}) = \frac{exp(\boldsymbol{\theta}\mathbf{h}(N))}{c(\boldsymbol{\theta})}$, where N is the adjacency matrix for the network, $\mathbf{h}(N)$ is the vector of network statistics, $\boldsymbol{\theta}$ is the vector of model coefficients, and $c(\boldsymbol{\theta}) = \sum_{i=1}^{\mathcal{N}} exp(\boldsymbol{\theta}(N_i))$, with $\mathcal{N}$ denoting the set of all possible networks with the same number of nodes. The vector of network statistics $\mathbf{h}$ can include structural terms (e.g. for reciprocity or triadic closure), node covariates, and dyad covariates. In TERGMs, the vector of network statistics $\mathbf{h}$ can additionally include dependencies on previously observed networks. In our case, we focus on a lag of one observation period (game round), so the model for network N at time t becomes: $P(N^t|N^{t-1}, \boldsymbol{\theta}) = \frac{exp(\boldsymbol{\theta}\mathbf{h}(N^t, N^{t-1}))}{c(\boldsymbol{\theta}, N^{t-1})}$.

The joint probability of observing all networks until period T can be then calculated by taking the product of the probabilities of the individual networks conditional on the others: $P(N^2, \dots, N^T|N^1, \boldsymbol{\theta}) = \prod_{t=2}^{T} P\,(N^t|N^{t-1}, \boldsymbol{\theta})$. We estimate our models using the R package *btergm*, which uses maximum pseudolikelihood with bootstrapped confidence intervals (Leifeld, Cranmer, and Desmarais 2018). The fit of the models was evaluated by goodness-of-fit diagnostic plots and plots that compare the network summary statistics of the empirical network with those of networks simulated by the model.

Since current implementations of the TERGM do not generalize to weighted networks, we fit the models on the non-weighted versions of the observed networks. As an alternative to the TERGM, we considered the stochastic actor-oriented model (SAOM), which is the other popular statistical model for longitudinally observed networks (Snijders, Bunt, and Steglich 2010). However, the SAOM assumes continuous time, strictly sequential decisions, and network changes between the observation periods (Leifeld and Cranmer 2019), all of which contradict our experimental setup.

References

Hanneke, Steve, Wenjie Fu, and Eric P. Xing. 2010. "Discrete Temporal Models of Social Networks." *Electron. J. Statist.* 4: 585–605. https://doi.org/10.1214/09-EJS548.

Leifeld, Philip, and Skyler J. Cranmer. 2019. "A Theoretical and Empirical Comparison of the Temporal Exponential Random Graph Model and the Stochastic Actor-Oriented Model." *Network Science* 7 (1): 20–51. https://doi.org/10.1017/nws.2018.26.

Leifeld, Philip, Skyler J. Cranmer, and Bruce A. Desmarais. 2018. "Temporal Exponential Random Graph Models with Btergm: Estimation and Bootstrap Confidence Intervals." *Journal of Statistical Software* 83 (6).

Snijders, Tom A.B., Gerhard G. van de Bunt, and Christian E.G. Steglich. 2010. "Introduction to Stochastic Actor-Based Models for Network Dynamics." *Social Networks* 32 (1): 44–60. https://doi.org/10.1016/j.socnet.2009.02.004.
