# Supplementary material for: Inequality and fairness with heterogeneous endowments
Source: PLoS One. 2022 Oct 31;17(10):e0276864. doi: 10.1371/journal.pone.0276864 (PMC9621428; doi:10.1371/journal.pone.0276864)
Supplement: S3 Text — (PDF) [file pone.0276864.s012.pdf]

Supplementary analyses

We investigate the relation between cooperativeness and outcomes to provide more detail about what drives the results in the different treatments (S7 Fig). On the one hand, we notice that the similar Gini coefficients in NI and E result from different group processes and dynamics. In NI, the dispersion of outcomes is driven by the low scores of the least endowed, while in E, it is driven by the higher unpredictability of scores for each endowment group (S8 Fig). In particular, in E, the highest endowed can achieve extreme success because they either exploit (never give to others) or cooperate unconditionally (always give to others); in contrast, in NI, the highest endowed gain the most from average levels of cooperation. On the other hand, we observe that the low Gini coefficients in the treatments with visible outcomes (O and EO) result from a similar phenomenon: cooperativeness does not affect the outcomes of those with lower endowments but it is the well-endowed unconditional cooperators who take a hit on their earnings. This is likely because they distribute their resources charitably, without establishing mutually beneficial reciprocal relations.
