# Supplementary material for: Inequality and fairness with heterogeneous endowments
Source: PLoS One. 2022 Oct 31;17(10):e0276864. doi: 10.1371/journal.pone.0276864 (PMC9621428; doi:10.1371/journal.pone.0276864)
Supplement: Attachment — Submitted filename: response_to_reviewers.pdf [file pone.0276864.s013.pdf]

Response to Reviewers

Editor

Journal Requirements:
When submitting your revision, we need you to address these additional requirements.
1. Please ensure that your manuscript meets PLOS ONE's style requirements, including those for file naming. The PLOS ONE style templates can be found at *https://journals.plos.org/plosone/s/file?id=wjVg/PLOSOne_formatting_sample_main_body.pdf* *and* *https://journals.plos.org/plosone/s/file?id=ba62/PLOSOne_formatting_sample_title_authors_affiliations.pdf*

We reformatted the manuscript and renamed the files to comply with the PLOS guidelines.

2. Thank you for stating the following in the Acknowledgments Section of your manuscript:
"This research was made possible through the generous support of the Volkswagen Foundation (Grant Ref. 92 173). The game development was partially supported by the Carlsberg Foundation (Grant no. CF16–0593). The funders had no role in the study design, data collection and analysis, decision to publish, or preparation of the manuscript. We would also like to acknowledge the Science at Home team for their constructive design input"
Please note that funding information should not appear in the Acknowledgments section or other areas of your manuscript. We will only publish funding information present in the Funding Statement section of the online submission form.
Please remove any funding-related text from the manuscript and let us know how you would like to update your Funding Statement. Currently, your Funding Statement reads as follows:
"This research was made possible through the generous support of the Volkswagen Foundation (Grant Ref. 92 173). The game development was partially supported by the Carlsberg Foundation (Grant no. CF16–0593). The funders had no role in the study design, data collection and analysis, decision to publish, or preparation of the manuscript."
Please include your amended statements within your cover letter; we will change the online submission form on your behalf.

We have removed the funding information from the Acknowledgements section. We do not require amendments to the Funding Statement.

3. We note that you have stated that you will provide repository information for your data at acceptance. Should your manuscript be accepted for publication, we will hold it until you provide the relevant accession numbers or DOIs necessary to access your data. If you wish to make changes to your Data Availability statement, please describe these changes in your cover letter and we will update your Data Availability statement to reflect the information you provide.

We continue working on formatting the data and analyses files to prepare them for publication. In order not to delay the re-review process, we will not update the Data Availability statement at this stage. However, we aim to have everything deposited at a public repository by the time the manuscript is accepted for publication.

> *4. Please include captions for your Supporting Information files at the end of your manuscript, and update any in-text citations to match accordingly. Please see our Supporting Information guidelines for more information: http://journals.plos.org/plosone/s/supporting-information*

We have included the captions and separate files for each figure, table, and text and updated the in-text-citations accordingly.

> *Additional Editor Comments:*
>
> *Considering the numerous constructive comments given by both reviewers, I invite the authors to address them improving the presentation of their study.*

We agree that the feedback we received has been most constructive and informative. We addressed most comments and suggestions and in the few cases when we deemed it unnecessary, we attempted to clarify and justify our decision. Below, we list each suggestion and how we responded to it.

<u>Reviewer #1</u>

> *This is a most interesting paper that synthesizes ideas from political theory, sociology, social psychology, and behavioral economics on fairness perceptions, sharing behavior, and related group-level outcomes of material inequality. It tests predictions using rich experimental data both at the group level (which I found most interesting) and at the individual level (using a sophisticated ERGM methodology). Overall, the design and data are well used and get at some long standing questions in novel ways. I have a number of questions and comments, which I hope are useful for the authors. I order my comments following their text:*
>
> *Abstract: It was not clear to me how the first half of the Abstract relates to the research questions and the manuscript's main thrust about visibility of endowments and outcomes. Also it remained unclear how the functional relationship between endowments and outcomes is investigated. Isn't this more of a classification exercise than an "investigation"? The main idea of the experiment also remained nebulous, isn't the "network cooperation experiment" about partner selection and sharing? Please clarify these points in the Abstract.*

We thank the reviewer for their extremely helpful and constructive feedback. In response to this particular point, we added a third sentence in the abstract to clarify the manuscript's focus and revised the description of the experiment to be more concrete and descriptive. As suggested, we also replaced "investigate" with "catalogue" to better represent the theoretical contribution.

Intro: The authors state that their work "contributes in several ways" (p. 3), but precisely what really is new, what new insights are yielded, and why this research is important has, in the current form, not been made crystal clear. Statements at the start of the Related Work section and in the Discussion section made it clearer, and I suggest moving these sentences into the Intro to carve out the contribution. Small point: On p. 3 the authors speak of combining "political theory, social psychology, and behavioral economics" and then they cite several sociology papers in the same paragraph.

We revised, expanded, split, and structured the relevant paragraph in the Introduction to explain better how our study stands out from and contributes to the literature. Regarding the citations on p.3, we used "social psychology" to refer to this work as it belongs to the social psychology subfield of sociology ("sociological social psychology"). However, in order to avoid confusion with "psychological" social psychology, which we do not cite, we replaced "social psychology" with "sociology" in the list of disciplines.

Theory section: The authors use a wide definition of what they call "endowments," including privileged social origin and luck (p. 5, 6). Then they posit that meritocracy is characterized by a linear mapping of endowments to outcomes. This contradicts the typical understanding of meritocracy as a state of the social world where rewards are determined by performance (merit as opposed to privilege and luck).

We have expanded and clarified how we use the term "meritocracy." As with "fairness," this is a term that has been repeatedly redefined and pulled apart by social critics and politicians and unfortunately, we are not able to capture all the nuances of the ongoing debate in this particular study. Nevertheless, our broad definition of endowments fits well within this debate and also aligns with our attempt to refrain from normative statements and positioning.

The authors may want to explain in a bit more detail the seminal work by Lynn, Podolny and Tao (2009) which they use to motivate their theorizing on dispersion and leveling; most importantly perhaps: what are the conditions (e.g. uncertainty) under which dispersion or leveling occurs. To what degree is this study an experimental test of Lynn et al.'s theoretical work? A drawback for the usefulness of Lynn et al.'s framework here is that the authors' experimental design does not allow for increasing-return processes (p. 20) that in my reading is crucial in Lynn et al.'s model to disconnect outputs (e.g. status) from inputs (e.g. quality).

We thank the reviewer for this suggestion, as it was also brought up by Reviewer 2. Our study does not concern social status and thus, uncertainty and social influence are not conditions we investigate. Rather, our study is inspired by Lynn and colleagues' idea that decoupling between x (quality for them, endowments more generally for us) and y (status for them, outcomes such as payoffs and income for us) can occur in two different ways. We recognize that our brief reference did not explain this link clearly. In response, we expanded the description of Lynn et al.'s contribution and now also explicitly explain how our study relates to theirs.

To be able to better follow the derivation of hypotheses, and the fact that the asserted micro-level mechanisms pertain to both the choice of interaction partners and the choice about giving that together bring about group outcomes, it would be helpful to learn about the experimental situation briefly already in the Intro. The descriptions of human behavior that lead up to each hypothesis sound overly deterministic while at times it is unclear whether hypotheses follow strictly from theoretical claims or from prior empirical results.

We expanded the paragraph before the Hypothesis section to describe the specific social interaction setting we consider and how it affects our expectations. The changes we made also attempted to address a comment by Reviewer 2 who suggested that we need to explain why we do not discuss outcomes that involve dispersion.

Materials and Methods: The gamified experimental setting has not been motivated in the front end of the paper. Is this something of extra methodological interest (e.g. increasing external validity), a specific contribution, or is it just about subject engagement? How does it affect comparability to prior lab research?

We thank the reviewer for encouraging us to emphasize this innovation and contribution. We added a new paragraph in the introduction that delineates the significance of the gamified experimental setting.

Cliff's delta is a non-standard measure, at least in large parts of the social sciences. What are its benefits over more common (non-parametric) measures and are the results on leveling robust to changing the measure?

We added a sentence that clarifies that Cliff's delta is actually linearly related to the more common Mann-Whitney *U* with the added advantage that it gives a scaled measure and direction of effect. For this reason, we do not deem it necessary to replicate the results with the Mann-Whitney *U*. Another possible measure for levelling, which is perhaps more familiar to social scientists than Cliff's delta, is Cohen's *d*. Cohen's *d* is another effect size measure that estimates the standardized mean difference theta = (mu1 – mu2) /(st dev), where st dev is pooled over two groups. However, the concept of levelling as we present it focuses on the existence of distinction and not so much on the extent of distinction (which we capture with the concept of dispersion). Hence, we do not consider Cohen's *d* suitable in this case.

Results: I value highly the focus of group-level outcomes over the determinants of individual sharing behavior. This is where the manuscript makes its strongest contribution. I wondered why there is no result plot paralleling Fig. 1 (or a linear correlation analysis) despite statements like "This indicates that, in the absence of information, a meritocratic system emerges in which endowments and outcomes are linearly related" (p. 21).

We added new Fig 2 which plots exactly this and include a short discussion of the observed patterns. We were already showing this information in the Supplementary Information (S2

Fig in the revised manuscript) but we absolutely agree that it is helpful to present a more explicit comparison with the patterns depicted in Fig 1.

In the context of the micro-level ERGM analysis more interpretations with regard to partner selection would be interesting. The observational (or qualitative) study surrounding Fig. 5 nicely illustrates and adds to what has been shown in the main results.

We would require additional clarification what the reviewer has in mind. We note that the experiment is set up so that "giving" is equivalent to "selecting a partner" because participants could unilaterally pick to whom to give and this was the only decision they had to make; there was no possibility to "select a partner" without giving to them. We discuss giving choices extensively and partner choice is implied in this discussion.

Reviewer #2

This manuscript introduces an experimental design to understand how individual interactions can produce patterns of group-level outcomes that refer to traditional fairness concepts, when the visibility of endowments and partial outcomes is manipulated. These group-level outcomes emerge as a function of complex individual interactions that are not necessarily linked to the group and, in this sense, they are emergent. The manuscript was very interesting and it presented some important findings. However, I also think that it needs more work and some clarifications for the manuscript to reach its full potential. I hope the following comments help the authors improve their work.

We thank the reviewer for their positive evaluation and constructive feedback. We incorporated all suggestions.

The authors describe several group-level outcomes in terms of dispersion and leveling, the combination of which produces traditional fairness concepts at the group level, such as "equality of opportunity" or "wealth concentration." I found this discussion of how dispersion and leveling produce different fairness systems very interesting and one of the main contributions of the manuscript. Nonetheless, the connection of dispersion and leveling to what Lynn and colleagues (2009) argue in their article is somewhat confusing. The reason is that Lynn et al (2009) discuss those mechanisms to explain how social status can deviate from underlying heterogeneity in quality (e.g., distribution of skills). It is unclear to me how social status connects to the dispersion or leveling of outcomes in this theoretical discussion, especially considering that the mechanisms discussed by Lynn et al (2009) are mostly driven by social influence and, in my understanding, there is no social influence manipulation in the design. How is social influence addressed in the experimental design and how it relates to the concepts discussed by Lynn et al? Alternatively, I also thought that the authors were not really interested in social status per se but they were inspired by Lynn et al (2009) to discuss different principles of fairness as if there were dispersion and leveling in outcomes, borrowing these concepts from Lynn et al. If this is the case, the authors need to make this connection clearer and perhaps place the

reference to Lynn et al on a footnote as status dynamics are not directly relevant for the manuscript's discussion about fairness.

We thank the reviewer for prodding us to clarify the relation to Lynn et al. In fact, Reviewer 1 made a similar request. Our study does not concern status, nor the scope conditions Lynn and colleagues investigate. Rather, we were mainly inspired by their idea that decoupling between X (quality, endowments) and Y (status, outcomes) could occur in two different ways. In response, we have expanded our description of Lynn et al.'s contribution and now explicitly outline how our study relates to their ideas.

Moreover, the manuscript makes the claim that when there is no information about outcomes and endowments, a meritocratic distribution of final outcomes takes place: those who have more get more and those who have less get less. This point is unclear because it really depends on the nature of the endowments and the authors say that they do not want to specify whether endowments correspond to an ascribed or achieved characteristic (p. 6) – although they also state in the conclusion that they restrict "the definition of endowments to ascribed characteristics only" (p. 30), which makes the statement about meritocracy even more puzzling. In my understanding of the findings, however, I think that only if endowments are understood as achieved, one can see how the no-information condition can produce a meritocratic system. In other situations, we would need to accept some relationships between endowments and outcomes as meritocratic when they are clearly non-meritocratic – e.g., if we assume that endowments are similar to inheritances. I think the argument about meritocracy needs some clarification at different parts on the manuscript, particularly when the nature of endowments is discussed.

We want to point out that the text on p.30 says "we **refrained from** restricting our definition of endowments to ascribed characteristics only," so there is no contradiction in the text regarding how we define endowments. Nevertheless, we completely agree with the reviewer's overall concern that the discussion of meritocracy deserves clarification; this point was raised by Reviewer 1 too. In the section "Fairness at the group level" we expanded and clarified how we use the term "meritocracy." We note that while the common association of meritocracy is with achieved endowments, the most prominent critique of the concept notes that what we tend to perceive as achieved characteristics is often inherited. The spirit of the study is to refrain from normative statements and positioning and hence, we would like to preserve the broader definition of endowments. Nevertheless, in addition to this explanation, we have added additional clarifications/qualifications any time meritocracy is mentioned, wherever possible.

In the conclusion, the authors suggest some policy implications when they say that outcomes should be visible in some social settings such as the workplace and the school. I presume this implication is because one of the findings is that the visibility of outcomes decreases dispersion (as shown in figure 2) and because this pattern is driven mostly by "inequity aversion" and "helping the poor" (as shown in figure 4). However, it is unclear from the findings how other social mechanisms may interact with the visibility of outcomes in the social settings for which these policy designs are suggested. For instance, one could imagine a situation where groups emerge based on performance and low-performers are ostracized by others, not integrated as

suggested by the findings. This restriction (i.e., the absence of coalition formation) may be a scope condition for a finding that was produced with no intergroup dynamics. This point deserves some discussion in connection with the proposed implications.

This is an excellent point and we added a separate paragraph mentioning it in the Discussion. We also added two closing sentences to bring it up again at the end of the paper.

In a similar vein, it was interesting to see that no hypothesis referred to the dispersion of outcomes directly, even though dispersion is probably the most similar scenario to current distributions of income and wealth in the world: for developed countries, there is extreme wealth concentration and for developing countries, there is extreme inequality, following the schema in figure 1. The authors preempt important differences with other studies that produce wealth concentration in the "Related work" section in the front end and then go back to these differences in the conclusion. I believe that it is also important to say something about this when the hypotheses are discussed because the absence of hypotheses that relate to dispersion is really an unsaid statement about these findings not being expected, given the mechanisms of reciprocity, inequity aversion, and charity.

We added a paragraph before introducing the hypotheses to describe the concrete interaction situation we consider and why dispersion is not relevant. We argue that we need increasing return processes where endowments produce outcomes that can be re-invested as resources to obtain wealth concentration and poverty. We note that most of the previous studies on inequality we refer to do not include such possibility and hence, they do not produce dispersion that is higher than the initial or underlying heterogeneity. One exception perhaps is Shirado et al. (2019) who obtain outcome dispersion that increases after the initial period in the condition where individuals with higher endowments have more interaction partners and these interaction partners tend to have more resources ("degree assortative networks with unequal resources"). We have included now a reference to what we believe is a more relevant example – a study by Gächter at el. in which gains from one round of the public goods game can be reinvested in the next round.

Also, the conclusion asserts that "our empirical results may not simply transfer to different types of social interactions, network dynamics, or information institutions" and the research by Nishi et al (2015) that relates to wealth concentration is then discussed as an instance of this limitation. I think that the conclusion would be stronger if the authors mention instead what types of interactions their findings are informative about, and perhaps only then make a reference to Nishi et al (2015) as part of the scope conditions of their design (rather than as a limitation).

As suggested, we rewrote the relevant paragraph to focus on the scope conditions of our study and only then bring Nishi et al. (2015) as an example.

A few other comments are the following. First, the main analysis in figures 2 and 3 are based on 10 data points for each experimental condition. I wonder if the authors can say something about this low-N problem, why we should not be concerned that

the statistically significant differences are not due to noise (plotting confidence intervals can be better than presenting p-values), and why they decided to put aside the temporal evolution of the groups in the main analysis (I see that figure C1 includes the temporal evolution of the games). This could have increased the sample size and open up the possibility to use other modeling strategies to account for the more complex correlational structure between the variables of interest.

We agree that presenting the temporal evolution of the dynamics is informative and hence, we have redone Figs 2 and 3 to show mean estimates with confidence intervals over time (and in turn removed the old Fig C1 from the supplementary information). Nevertheless, we chose not to change the statistical tests, which are conducted over the final scores at the end of the game. In the last paragraph at the end of the section "Testing for dispersion and levelling," we added text explaining that we chose to trade off the power of temporal analysis for a simpler modelling strategy that considers the "equilibrium" state only. Overall, the new figures clearly show that the results would not change in either case and hence, we opted for the simpler and more conservative approach. We would also like to note that N=10 per treatment condition is not unusual since experiments with synchronous interaction in large groups tend to be quite expensive and difficult to conduct (for example, Nishi et al. 2015, Shirado et al. 2019, Melamed et al. 2022 also use N=10 per treatment condition). The manuscript already presents calculations that suggest that the power of the study is sufficient to detect meaningful effect sizes. Given the consistent trends and reasonable power, we do not consider that our low-N analysis poses a problem and we are confident in the findings.

References:

Melamed, D., Simpson, B., Montgomery, B., & Patel, V. (2022). Inequality and cooperation in social networks. *Scientific Reports*, 12(1), 6789.
Nishi, A., Shirado, H., Rand, D. G., & Christakis, N. A. (2015). Inequality and visibility of wealth in experimental social networks. *Nature*, 526(7573), 426–429.
Shirado, H., Iosifidis, G., & Christakis, N. A. (2019). Assortative mixing and resource inequality enhance collective welfare in sharing networks. *Proceedings of the National Academy of Sciences*, 116(45), 22442–22444.

Second, it seems to me that endowments can be inferred in the O condition, similarly to what happens in real life where we may have clear information about wealth or income and no information about endowments but try to infer what the distribution of endowments is. This could explain why this condition is so similar to the EO condition in several of the findings presented on the manuscript, including individual behaviors. Is this inference possible? Third,

We agree that participants may have attempted to infer the endowments when only the outcomes were visible but we do not believe this had a prominent effect on their behavior. We note that the O condition is similar to the EO condition only in terms of the effects of visible outcomes on dispersion; it is not similar to either to the E condition or the EO condition in terms of equality of opportunity, which we show is the result of visible endowments. But even so, we feel that such potential inference is not a conceptual flaw nor a validity problem because it approximates real-life situations, where individuals often make assumptions about quality based on success.

Minor comments

• I see that the authors talk about "the dispersion of the outcome" in the appendix to refer to the variance of the outcome (e.g., "the outcomes are more/most dispersed…" in figures C4 and C5 or "the dispersion of the outcomes is driven by the low scores…" in p. 8, Appendix C). I found this language confusing as they introduce the theoretical concept of "dispersion" in regard to fairness and inequality in the front end.

We recognize that a better distinction between how we use the terms dispersion is necessary. We have expanded the paragraph describing the Gini coefficient to describe the precise relationship between the Gini as a measure of statistical dispersion and our concept of dispersion as an indicator of inequality. Throughout the text, we distinguish between the two by using "dispersion" for our concept and "statistical dispersion" for what we measure.

• In page 24, the authors say that they manually coded answers to the question about individual strategies. Can they say a bit more about how this task was actually performed? For instance, is there intercoder reliability?

We updated the text to explain how we completed the coding task, to report intercoder reliability, and to clarify how we resolved coding conflicts.
