# Supplementary material for: Inequality and fairness with heterogeneous endowments
Source: PLoS One. 2022 Oct 31;17(10):e0276864. doi: 10.1371/journal.pone.0276864 (PMC9621428; doi:10.1371/journal.pone.0276864)
Supplement: Attachment — Submitted filename: response_to_reviewers.docx [file pone.0276864.s014.docx]

**Response to Reviewers**

**Editor**

*Please review your reference list to ensure that it is complete and correct. If you have cited papers that have been retracted, please include the rationale for doing so in the manuscript text, or remove these references and replace them with relevant current references. Any changes to the reference list should be mentioned in the rebuttal letter that accompanies your revised manuscript. If you need to cite a retracted article, indicate the article’s retracted status in the References list and also include a citation and full reference for the retraction notice.*

We reviewed the references and added two new ones to the References list that we had overlooked before. We also fixed typos for several in-text references that made it appear that we refer to other work. We are not aware of any papers among the ones we cite that have been retracted. If the journal has that knowledge, we would be thankful if they point us to the specific work so that we can reconsider including it.

*Considering both reviewers' positive evaluation, the manuscript can be now considered for publication after a minor revision to address Reviewer 2's last remarks. In particular, there are some parts that can still improve the presentation of the study. In case the author satisfactorily addresses these comments an additional round of revision won't be needed before proceeding for publication.*

We address the comments by Reviewer 2 below. We would also like to note that we updated the Data Availability Statement for the manuscript, where we now point to the public repository storing the data and analysis scripts for the study. Finally, we also added a sentence in the acknowledgement to thank the two anonymous reviewers. The reviewers’ engagement with and contribution to this work has been outstanding and invaluable.

**Reviewer 2**

*I appreciate all the work that went into preparing this revision, and I commend the authors for this version. Most of my previous concerns were addressed and clarified. I also like how the introduction reads now: it states clearly what the question is and what the contributions are. However, a few concerns remain. I leave my comments to the authors below, which I hope are helpful to the authors.*

As before, we are grateful to the reviewer for their careful reading of our work and most constructive feedback. We describe how we addressed each specific point below.

*• The text is much clearer about the difference between dispersion and leveling. However, the new version makes me think that the reference to Lynn et al. (2009) is unnecessary. If anything, it adds more confusion to the authors’ explanation. While the concept of dispersion used in the manuscript is similar to the use in Lynn et al., the concept of leveling does not entirely match the use of ‘rank reordering’ in Lynn et al. I think the slopes in figure 1 should be negative to reflect the concept of rank reordering more appropriately. Since this comparison is not crucial for the manuscript’s argument, I would suggest that the authors either eliminate that paragraph or leave it in a footnote for readers curious about the parallels.*

We agree with the reviewer that the new expanded discussion is somewhat tangential and likely confusing to the general reader. Since *PLoS ONE* does not allow footnotes, we decided to delete the paragraph under question and remove the reference.

*• The authors define poverty and wealth in terms of dispersion in the front end, not in terms of Gini. Consequently, the authors should not conclude that the treatments do not produce poverty or wealth concentration because the Gini coefficients are low. They say this happens because their interaction situation model leads to lower Gini coefficients by design (p. 25). It would be better to state that the treatments do not produce poverty or wealth concentration because no condition leads to steeper slopes (as shown in fig. 2).*

We recognize that the new Fig 2 we added in the previous revision may have somewhat obfuscated the distinction between the conceptualization and the operationalizations. We use the Gini coefficient to operationalize the concept of dispersion. We cannot use the slope because the slope of the dashed line in Fig 1 (the expected ideal proportional relationship between endowments and outcomes) cannot be quantified a priori. Fig 1 attempts to depict the concepts of dispersion and levelling with a single approach but we quantify these with two different measures (Gini coefficient and Cliff’s delta). The axes in Fig 1 have no units and the slope of the dashed line is arbitrary. Similarly, the results in Fig 2 are centered on the outcomes of those with average endowment and present the others’ outcomes only in relevant terms. Fig 2 says nothing about what the expected/baseline slope is and hence, whether the empirical slopes we observe are “steeper.” The make this judgement quantitatively, we operationalize dispersion with the Gini coefficient and use the Gini of endowments as the baseline for our specific context. In other contexts, different baselines could be used, and yet our conceptualization of fairness would remain.

To clarify the distinction between conceptualization and operationalization, we have added additional explanation at several points in the relevant sections. We replaced “quantify” in the Introduction with “compare” and “juxtapose” and we added a comment about the slope. Additionally, we expanded the first paragraph of the section “Testing for dispersion and leveling” to emphasize the link and distinction between the concepts and their operationalization. Finally, we also added additional text in the discussion of Fig 2 to clarify why reading it directly may be misleading.

*• I still have questions about the concept of meritocracy. I understand what the authors are trying to do, and I think that stating explicitly how they are using meritocracy in this setting helps the reader offset some of the implicit notions they have about the notion of meritocracy when reading the manuscript. The critical problem is that some deviations from the meritocratic scenario imply different uses of the concept of ‘endowment.’ For instance, when the authors describe equality of opportunity, they say it means that “individuals should not be limited by characteristics ascribed at birth.” Hence, even if the reader is willing to concede that endowments can refer to ascribed or achieved characteristics, we are forced to think about endowments as ascribed—not achieved— when we compare the meritocratic condition with equality of opportunity. In the E condition, the Cliff’s delta statistic remains low (in fig. 4) because participants likely interpret endowments as ascribed (given that they are exogenously assigned). Why would participants give charitably to the least endowed (as stated in the conclusion; p. 33) if they do not perceive endowments as ascribed? In short, although it is clear conceptually why endowments could be regarded as ascribed or achieved, the experimental design forces participants (and readers) to understand endowments only as ascribed.*

This is a valid point – the experiment indeed models endowments as ascribed and we should have pointed this out in the discussion of the research limitations. We have done so now on p.32. We thank the reviewer for bringing it to our attention.

*• In figure 2, the authors state that “when outcomes are visible, they end up less equalized and less variable” (p. 23). I only see this for participants with 2 and 4 endowments, however. The score difference for the most endowed is highly variable, especially in the O condition. Am I missing something here?*

The description of Fig. 2 is qualitative and intends to foreshadow the quantitative results that follow. Indeed, the variability is visibly smaller for endowments 2 and 4. For endowment 6, the O condition has visibly less dispersed outcomes in comparison to the E condition but not in comparison to the NI condition. The reviewer’s observations are correct but there is no contradiction between the visualization and the interpretation; simply, the trend is not visible enough. In response , we have slightly modified the language in the sentence to convey the speculative nature of the statement and emphasize that the actual statistical evidence is yet to be presented.

*• Moreover, I found it very interesting to compare the results in terms of dispersion and leveling (figs. 3 and 4) with the distribution of scores across endowments in fig. 2. I believe that crossing the information from these figures renders a couple of insights that are not highlighted enough in the manuscript. For instance, in the conclusion, the authors say that the visibility of outcomes leads to equality due to lower Gini (p. 32 and p. 33). But this understates the shape of final scores in figure 2 and the variability of Cliff’s delta between conditions O and EO. Indeed, fig. 2 suggests that both conditions resemble equality of opportunity. Fig. 4 shows that this form of equality in condition EO occurs because the lower class (with two endowments) becomes similar to the middle class (with four endowments) regarding how opportunities map to outcomes. In condition O, equality of opportunity is also achieved, but endowments seem more consequential for the lower class (i.e., there is less mobility), as observed in fig. 4. This comparison makes me think that the visibility of outcomes and the visibility of endowments are not as different as implied in the conclusion (e.g., p. 33.). More generally, the addition of fig. 2 makes the reader wonder whether the findings for group-level fairness can be described in terms of the fairness concepts represented in fig. 1.*

We recognize the reviewer’s point that the distinction between Endowment levels 2 and 4 in the O and EO conditions does not appear salient in Fig.2 and thus, seems to contradict the claims based on Fig.4. However, as we explained under the point about the Gini above, we would like to keep an explicit distinction between conceptualization and operationalization and between visualizations and statistical tests. Fig 2 visualizes the results but does not allow us to test them statistically. Figs 3 and 4 break down the two distinct concepts of dispersion and leveling we started with, operationalize them, and statistically test them against baselines and among treatments. We hope that the clarifications we added in the text in response to the Gini point above serve to emphasize this distinction and dispel confusion.
